# What viruses can teach us about the human immune system

**DOI:** 10.1371/journal.ppat.1006364

**Published:** 2017-07-13

**Authors:** Michaela U. Gack

**Affiliations:** Department of Microbiology, University of Chicago, Chicago, Illinois, United States of America; The Fox Chase Cancer Center, UNITED STATES

The first and most critical step in a host’s response to viral infection is the recognition of the viral invader by the immune system. This task is carried out by a repertoire of sensor molecules, found in most organisms, that recognize features that are unique to the virus and not found in the host, such as viral double-stranded RNA. The principle that hosts are able to detect specific pathogen-derived patterns was first proposed by Charles Janeway in 1989, and since then, a number of innate immune receptors including several viral RNA and DNA sensors have been identified. These sensor proteins are part of the so-called innate immune system, the branch of the immune system that protects the host from viruses and other pathogens immediately after infection, and thus acts much earlier than the other branch of the immune system, the adaptive immune response. Following virus recognition, these sensors initiate signaling cascades that result in the induction of an antiviral program characterized by the up-regulated expression of many antiviral or inflammation-inducing molecules such as cytokines like interferons.

It was an exciting time in the field of innate immunity when I started my PhD studies in 2005. Two new intracellular viral RNA sensors called retinoic acid-inducible gene-I (RIG-I) and melanoma differentiation-associated gene 5 (MDA5) had just been discovered. Prior to this, study of the innate immune system had focused extensively on membrane-bound receptors, such as the Toll-like receptors, which are primarily found in specialized immune cells. The discovery of RIG-I and MDA5 was exciting because these cytoplasmic sensors were found to be expressed in practically all cells in the human body, indicating that even nonimmune cells have the ability to sense viral invaders and to launch an innate immune response. We now know that RIG-I and MDA5 play a critical role in the detection of a broad range of RNA viruses, including influenza virus, dengue virus, and hepatitis C virus.

When I was starting my PhD studies at Harvard University, the antiviral function of RIG-I had just been discovered, but how this sensor worked and signaled to initiate an antiviral program was unknown. Using affinity purification of the RIG-I protein and mass spectrometry analysis, I discovered that RIG-I is modified with ubiquitin molecules, a process known as ubiquitination. While most cellular proteins undergo ubiquitination, which often marks them for proteasomal degradation, I found that RIG-I was modified by a quite unique type of polyubiquitin that did not lead to proteasomal degradation of RIG-I but instead activated RIG-I to signal downstream. Another round of RIG-I purification from human cells and mass spectrometry identified the enzyme responsible for RIG-I ubiquitination and activation: tripartite motif protein 25 (TRIM25). TRIM25 belongs to the TRIM protein family of ubiquitin E3 ligases with about 70 members in humans. Our work showed for the first time that a TRIM molecule can directly activate a viral pathogen sensor through nondegradative ubiquitination and thereby promote antiviral cytokine production. Over the past several years, we have learned amazing things about the TRIM protein family, whose members can act as antiviral restriction factors by binding to viral components or, in a similar fashion as TRIM25, regulate innate immune responses. Today, about 10 years later, an immunomodulatory role for many other TRIM proteins has been identified, strengthening the concept that TRIM proteins are key regulators of antiviral and proinflammatory responses. Even more exciting, completely new functions for TRIM proteins have been more recently identified, for example, their roles in virus-induced autophagy, and I believe there is still much more to learn about this fascinating group of proteins. In regard to translational research, I am confident that mechanistic insights into how TRIM proteins exert their antiviral activity will reveal ways to manipulate specific TRIM proteins for clinical interventions for infectious diseases or situations in which our immune system has gone awry, such as autoimmune disorders.

Millions of years of battle between viruses and hosts have facilitated the coevolution of the 2 factions, and viruses have evolved sophisticated mechanisms to block the human immune system. As an undergraduate student at the University of Erlangen-Nuremberg, Germany, I worked on gamma-herpesviruses and their strategies to manipulate signal transduction in lymphocytes. As I learned more in graduate school about how the innate immune system defends the cell against viruses, my background in virology prompted me to also look at the other side of the coin: how viruses counteract these host defenses. After uncovering the antiviral function of TRIM25 in the RIG-I–mediated immune response, I asked the question of whether some viruses are able to block RIG-I and TRIM25 to evade detection by the innate immune surveillance machinery. Other groups had demonstrated that influenza A viruses are able to effectively suppress interferon induction via the viral protein NS1, but how this protein inhibited the sensor RIG-I was largely unknown. We discovered that NS1 antagonizes RIG-I signaling by interacting with TRIM25 and blocking its ability to ubiquitinate RIG-I.

Studying how viruses dysregulate the immune system has provided us with important insight into how the immune system works, and in turn, this knowledge may allow us to manipulate the innate immune response for the development of therapies. In my own laboratory, first at Harvard and more recently at the University of Chicago, we are employing a combination of approaches, including proteomics, biochemistry, and cell biology, to unravel the mysteries of how innate immunity is initiated and how viruses antagonize this response. While mechanistic detail is at the heart of our studies, I believe it is important to always keep the big picture in mind. Key molecules in innate immunity have repeatedly been shown to be major targets of viral antagonism, and the ultimate goals of our studies are to find ways of attenuating viruses by targeting their immune escape mechanisms and to develop means of boosting the human immune system using what we know about innate immune signaling.

An understanding of the molecular mechanisms behind the activation of innate immunity could potentially be translated into the ability to boost our immune system in a way that is broadly applicable to combating many different viruses instead of just one. I believe this strategy could be important in light of recent viral outbreaks that have demonstrated that the advent of new or reemerging viruses is unpredictable, and, consequently, it might be difficult to develop specific antiviral therapies for each newly emergent viral pathogen, at least in a timely manner. At the same time, our work on the immune evasion mechanisms employed by influenza virus and, more recently, dengue virus has shown that eliminating a critical immune evasion strategy of these viruses results in crippled, attenuated viruses that are immunogenic. Our work over the years has reinforced my belief that basic research into the fundamental molecular mechanisms of innate immune signaling and viral evasion is critical for the rational design of new vaccines and antiviral therapies for combating emerging viruses and also viruses that cause persistent infections. Approaching our research questions from both the host and virus perspectives has been illuminating, and we will continue to look at both sides of the equation for insight into the innate immune system.

**Image 1:**
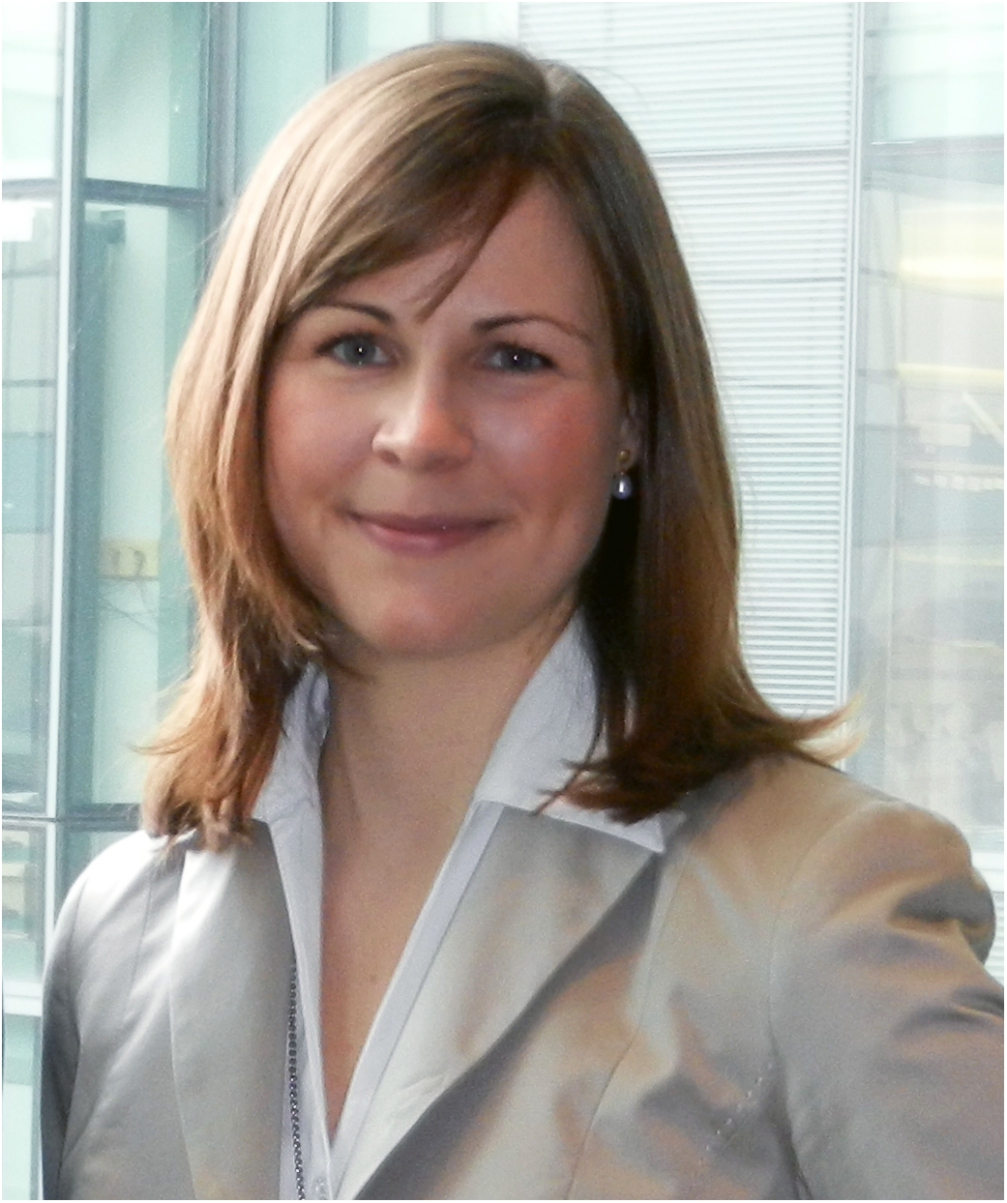
Michaela Gack.

